# A Novel IncA/C1 Group Conjugative Plasmid, Encoding VIM-1 Metallo-Beta-Lactamase, Mediates the Acquisition of Carbapenem Resistance in ST104 *Klebsiella pneumoniae* Isolates from Neonates in the Intensive Care Unit of V. Monaldi Hospital in Naples

**DOI:** 10.3389/fmicb.2017.02135

**Published:** 2017-11-03

**Authors:** Eliana P. Esposito, Stefano Gaiarsa, Mariateresa Del Franco, Valeria Crivaro, Mariano Bernardo, Susanna Cuccurullo, Francesca Pennino, Maria Triassi, Piero Marone, Davide Sassera, Raffaele Zarrilli

**Affiliations:** ^1^Department of Public Health, University of Naples ‘Federico II’, Naples, Italy; ^2^Department of Bioscience, University of Milan, Milan, Italy; ^3^Microbiology and Virology Unit, Fondazione Istituto di Ricovero e Cura a Carattere Scientifico (IRCCS) Policlinico San Matteo, Pavia, Italy; ^4^Azienda Ospedaliera di Rilievo Nazionale (AORN) dei Colli, V. Monaldi Hospital, Naples, Italy; ^5^Department of Biology and Biotechnologies, University of Pavia, Pavia, Italy; ^6^Centro di Inngegneria Genetica (CEINGE) Biotecnologie Avanzate, Naples, Italy

**Keywords:** carbapenemase producing *Klebsiella pneumoniae*, VIM-1 carbapenemase, IncA/C plasmid, horizontal gene transfer, neonatal intensive care unit

## Abstract

The emergence of carbapenemase producing Enterobacteriaceae has raised major public health concern. The aim of this study was to investigate the molecular epidemiology and the mechanism of carbapenem resistance acquisition of multidrug-resistant *Klebsiella pneumoniae* isolates from 20 neonates in the neonatal intensive care unit (NICU) of the V. Monaldi Hospital in Naples, Italy, from April 2015 to March 2016. Genotype analysis by pulsed-field gel electrophoresis (PFGE) and multi-locus sequence typing (MLST) identified PFGE type A and subtypes A1 and A2 in 17, 2, and 1 isolates, respectively, and assigned all isolates to sequence type (ST) 104. *K. pneumoniae* isolates were resistant to all classes of β-lactams including carbapenems, fosfomycin, gentamicin, and trimethoprim–sulfamethoxazole, but susceptible to quinolones, amikacin, and colistin. Conjugation experiments demonstrated that resistance to third-generation cephems and imipenem could be transferred along with an IncA/C plasmid containing the extended spectrum β-lactamase *bla*_SHV -12_ and carbapenem-hydrolyzing metallo-β-lactamase *bla*_V IM-1_ genes. The plasmid that we called pIncAC_KP4898 was 156,252 bp in size and included a typical IncA/C backbone, which was assigned to ST12 and core genome (cg) ST12.1 using the IncA/C plasmid MLST (PMLST) scheme. pIncAC_KP4898 showed a mosaic structure with *bla*_V IM-1_ into a class I integron, *bla*_SHV -12_ flanked by IS6 elements, a mercury resistance and a macrolide 2′-phosphotransferase clusters, *ant(3″), aph(3″), aacA4, qnrA1, sul1*, and *dfrA14* conferring resistance to aminoglycosides, quinolones, sulfonamides, and trimethoprim, respectively, several genes predicted to encode transfer functions and proteins involved in DNA transposition. The acquisition of pIncAC_KP4898 carrying *bla*_V IM-1_ and *bla*_SHV -12_ contributed to the spread of ST104 *K. pneumoniae* in the NICU of V. Monaldi Hospital in Naples.

## Introduction

The spread of carbapenem-resistant Enterobacteriaceae (CRE) has increased globally and these strains have become endemic in several countries including Italy. CRE may colonize or infect patients both in the hospital and in the community setting (Nordmann and Poirel, 2014; Del Franco et al., 2015; Onori et al., 2015; Pitout et al., 2015; Conte et al., 2016; Logan and Weinstein, 2017). The prevalence of CRE infections is increasing among children and neonates also (Logan, 2012; Logan et al., 2015; Zhu et al., 2015).

The international spread of CRE is primarily due to clonal expansion of isolates belonging to *Klebsiella pneumoniae* and *Escherichia coli* epidemic clonal lineages (Bialek-Davenet et al., 2014; Nordmann and Poirel, 2014; Del Franco et al., 2015; Gaiarsa et al., 2015; Pitout et al., 2015; Conte et al., 2016; Logan and Weinstein, 2017).

Additionally, CRE dissemination is contributed by horizontal gene transfer of carbapenemase genes carried by transposons and plasmids (Pitout et al., 2015; Logan and Weinstein, 2017). Class B metallo-β-lactamases (MBLs) (IMP, VIM, NDM), class A (KPC) or class D (OXA-48) serine carbapenemases have been isolated worldwide (Nordmann and Poirel, 2014; Del Franco et al., 2015; Pitout et al., 2015; Conte et al., 2016; Giani et al., 2017; Grundmann et al., 2017; Khan et al., 2017; Logan and Weinstein, 2017; Matsumura et al., 2017). Of these, MBLs show increasing clinical relevance because they cannot be neutralized by the available β-lactamase inhibitors and are able to horizontally disseminate via mobile genetic elements. Among acquired MBLs, VIM- and NDM-type enzymes are those having the widest geographical distribution and range of bacterial hosts (Di Pilato et al., 2014; Peirano et al., 2014; Zurfluh et al., 2015; Khan et al., 2017; Matsumura et al., 2017).

Because intestinal carriage of CRE is an important source of transmission, guidelines have been established worldwide to monitor and isolate CRE carriers in health care facilities (Nordmann and Poirel, 2014; Viau et al., 2016).

The aim of this study was to analyze the molecular epidemiology of VIM-1 producing *K. pneumoniae* isolates from intestinal carriers in neonatal intensive care unit (NICU) of an Italian hospital in Naples and to characterize the structure of the conjugative plasmid, which mediates the horizontal transfer of carbapenem resistance.

## Materials and Methods

### Setting

The NICU of V. Monaldi Hospital in Naples is a tertiary care level NICU and consists of three rooms and 16 cot spaces with a 1:2–1:3 nurses/neonates ratio. The NICU serves approximately 260 admissions per year and admits exclusively babies from the regional Newborn Emergency Transport Service or Territorial Emergency Service and through the transfer from the internal departments of Pediatric Cardiology and Pediatric Heart Surgery. In case of necessity, the department performs repeated hospitalizations for some particular types of newborns (lower birth weight, heart disease, etc.). Active patient-based surveillance of healthcare-associated infections on neonates with >2 days NICU stay is performed as previously described (Horan et al., 2008; Crivaro et al., 2015). Surveillance of CRE in V. Monaldi Hospital, based on rectal swabs at hospital admission, is performed according to the guidelines of European Centre for Disease Prevention and Control [ECDC] (2012) and the Ministero della Salute (2013), Italy.

### Bacterial Strains and Microbiological Methods

*Klebsiella pneumoniae* isolates were identified using the Vitek 2 automatic system and the ID-GNB card according to the manufacturer’s instructions (bioMérieux, Marcy l’Etoile, France) as previously described (Del Franco et al., 2015).

### Antimicrobial Susceptibility Testing

Carbapenem resistance of Enterobacteriaceae was screened using the meropenem disk alone as previously described (Pournaras et al., 2013). Identification of MBL activity was performed using imipenem + EDTA 15 + 750 μg combined disk method (ROSCO Diagnostica A/S, Taastrup, Denmark). Susceptibility tests were performed using the Vitek 2 system and the AST-GN card (bioMérieux, Marcy l’Etoile, France); carbapenem and colistin susceptibility were evaluated by broth microdilution in Mueller–Hinton broth II (MHBII) according to Clinical and Laboratory Standards Institute guidelines (CLSI, 2015). Breakpoints values were those recommended by the EUCAST (2016).

### Molecular Analysis of Antimicrobial Resistance Genes

Characterization of β-lactamase genes was performed as previously described (Poirel et al., 2011). Two multiplex PCRs were set up, the reaction no. 1 including detection of *bla*_KPC_ and *bla*_OXA-48-like_ and the reaction no. 2 including detection of *bla*_IMP_, *bla*_V IM_, and *bla*_NDM_. The following thermal cycling conditions were used: 3 min at 94°C and 35 cycles of amplification consisting of 1 min at 94°C, 1 min at 57°C, and 1 min at 72°c with 5 min at 72°C for the final extension. PCR products were analyzed by electrophoresis in a 1.8% agarose gel stained with ethidium bromide. The following strains were used as positive quality controls: *Acinetobacter baumannii* AC 54/97 (Riccio et al., 2000), *A. baumannii* 161/07 (Bonnin et al., 2012), *K. pneumoniae* D001 (Pournaras et al., 2013), *K. pneumoniae* 1, *K. pneumoniae* 2, and *E. coli* 6 (Del Franco et al., 2015) for *bla*_IMP-2_, *bla*_NDM-1_, *bla*_OXA-48_, *bla*_KPC-2_, *bla*_KPC-3_, and *bla*_V IM-1_ carbapenemase genes, respectively. The full-length alleles of bla_V IM_ were amplified using primers 5′VIM1 and 3′VIM1 shown in Supplementary Table S1. Sanger DNA sequencing and identification of deduced amino acid sequences were performed as previously described (Del Franco et al., 2015).

### PFGE Typing and Dendrogram Analysis

*Klebsiella pneumoniae* isolates were genotyped by *Xba*I DNA macrorestriction, pulsed-field gel electrophoresis (PFGE) and dendrogram analysis as described previously (Del Franco et al., 2015).

### MLST Analysis

*Klebsiella pneumoniae* isolates were typed using the Institut Pasteur’s MLST (multi-locus sequence typing) scheme (Diancourt et al., 2005) and primes and PCR conditions available at http://bigsdb.pasteur.fr/klebsiella/primers_used.html eBURST analysis of profiles available at http://bigsdb.pasteur.fr/perl/bigsdb/bigsdb.pl?db=pubmlst_klebsiella_seqdef_public&page=profiles was performed as described previously (Feil et al., 2004). Minimum **s**panning trees of sequence types (STs) were built by Phyloviz using the goeBURST algorithm (Francisco et al., 2012).

### Conjugative Transfer of Carbapenem Resistance and Plasmid Typing

Filter mating experiments were performed using sodium azide resistant *E. coli* J53 (Jacoby and Han, 1996) as recipient strain in the presence of either 5 μg/ml imipenem and 100 mg/l sodium azide or 50 μg/ml ampicillin and 100 μg/ml sodium azide. The frequency of transfer was calculated as the number of transconjugants divided by the number of surviving recipients as previously described (Zarrilli et al., 2005). Plasmids typing was performed by PCR-based replicon typing (PBRT) kit (Diatheva s.r.l., Fano, Italy) using previously described primers and conditions (Carattoli et al., 2005, 2006).

### Whole-Genome Sequencing and Plasmid Reconstruction

DNA was extracted from the *K. pneumoniae* parental strain, and from the *E. coli* strain before and after transconjugation using a DNeasy Blood & Tissue Kit according to the manufacturer’s instructions (Qiagen, Milan, Italy). Whole genome sequencing was performed using an Illumina Miseq platform with a 2 by 250 paired-end run after Nextera XT paired-end library preparation. Sequencing reads from the *K. pneumoniae* parental strain and from the *E. coli* strain before the transconjugation obtained in this study were assembled using the software SPAdes (Bankevich et al., 2012) with accurate settings.

The BIGSdb genome database software (Jolley and Maiden, 2010) was used in the BIGSdb-Kp database^1^ to identify genes associated with virulence, heavy metal and drug resistance in *K. pneumoniae* 4898 genome sequence. *In Silico* detection of plasmids was performed using PlasmidFinder Web tool at https://cge.cbs.dtu.dk/services/PlasmidFinder/ as previously described (Carattoli et al., 2014). Sequencing reads of the *E. coli* transconjugant strain containing the plasmid were mapped to the assembled genome of the same *E. coli* sequenced prior to transconjugation, using the mapping software Bowtie2 (Langmead and Salzberg, 2012). All non-mapping reads were extracted and assembled using SPAdes (Bankevich et al., 2012) with accurate settings. Presence of the obtained contigs in the original *K. pneumoniae* genome was verified by blast-searching followed by manual analysis, i.e., all the newly obtained contigs that aligned with the *K. pneumoniae* assembly with an identity over 95% were kept. The co-linearity of the contigs was assessed using Bandage tool for visualizing assembly graphs (Wick et al., 2015). The connections indicated by Bandage were used as starting point for finishing experiments by PCR and Sanger sequencing to bridge the ends of contigs (see Supplementary Table S1 for a complete list of PCR primers used).

### Plasmid *in Silico* Typing and Annotation

Plasmid MLST (PMLST) and core genome PMLST (cgPMLST) analysis of Inc A/C plasmid profiles available at https://pubmlst.org/bigsdb?db=pubmlst_plasmid_seqdef was performed as previously described (Hancock et al., 2017). The 28 IncA/C conserved genes from each PMLST profile were aligned using Muscle (Edgar, 2004). Unreliable positions were removed from each alignment using Gblocks (Castresana, 2000). All alignments were concatenated and used as input for a maximum likelihood phylogenetic analysis, which was performed with the software PhyML 3.0 (Guindon et al., 2010) using the GTR substitution model. PMLST minimum spanning trees were built by Phyloviz (Francisco et al., 2012). The gene annotation of the plasmid was performed using the software Prokka (Seemann, 2014) followed by accurate manual control, based on blast-searches against the nr-protein database. Inverted repeats were identified manually, based on the sequence and patterns found in Hancock (AAC 2017).

### Nucleotide Sequence Accession Numbers

The genome sequences of *K. pneumoniae* 4898 and plasmid pIncAC-KP4898 have been deposited in the GenBank nucleotide database under accession numbers FWYI00000000.1 and KY882285, respectively.

### Ethics Statement

The study has been evaluated by the local Ethics committee (Comitato Etico Università degli Studi della Campania “Luigi Vanvitelli” AOU “Luigi Vanvitelli” – AORN “Ospedali dei Colli”) (protocol number 421/2017). Because the patients included in the study were anonymized, no written informed consent was required.

## Results

### Epidemiology of CR *K. pneumoniae* in the NICU and Infection Control Measures

**FIGURE 1:**
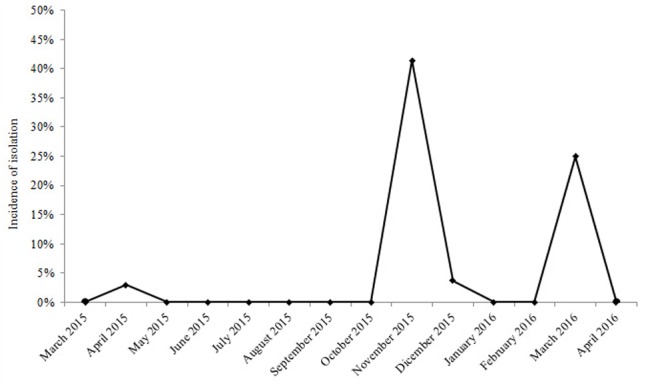
Incidence of isolation of VIM-1 carbapenemase producing ST104 *Klebsiella pneumoniae* from neonates in the NICU of V. Monaldi Hospital in Naples, Italy, during 2015 and 2016.

The emergence of carbapenem-resistant (CR) *K. pneumoniae* was observed in the NICU of V. Monaldi Hospital from October 2015 to March 2016, when CR *K. pneumoniae* were isolated from rectal swabs of 19 neonates into two consecutive clusters. Only one sporadic CR *K. pneumoniae* isolate was obtained from a rectal swab of a neonate in the NICU of the hospital during the previous 22 months, while CRE were occasionally isolated from rectal swabs and other clinical specimens of adult patients in other wards of the hospital (**Figure 1** and data not shown). Immediately following the first isolation of CR *K. pneumoniae*, a multimodal infection control program was implemented in the NICU, which included: weekly or biweekly screening for CRE at rectal swab of hospitalized neonates, increased frequency of environmental cleaning using chloride derivatives at 1100 ppm, reinforce handwashing compliance before and after patient contact, and daily visits to the ward by the hospital Infection Control Nurse. Colonized neonates were isolated either structurally or following strict adherence to contact precautions and staff cohorting was performed. During the two clusters, the ward was temporarily closed to external admissions. Environmental microbiological investigation of room surfaces, equipment, and staff hands failed to identify sources or reservoirs of CR *K. pneumoniae*. Neonates’ mothers were not screened for the presence of CR *K. pneumoniae*. Because all neonates with CR *K. pneumoniae* isolation did not show signs of infection, they did not receive antimicrobial therapy against CR *K. pneumoniae* and were not eradicated before discharge. The cluster ended on March 2016 when the last CR *K. pneumoniae* intestinal carrier neonate was discharged from the ward (**Figure 1**).

### Antimicrobial Susceptibility Testing and Characterization of Carbapenemase Genes in CR *K. pneumoniae* Isolates

**FIGURE 2:**
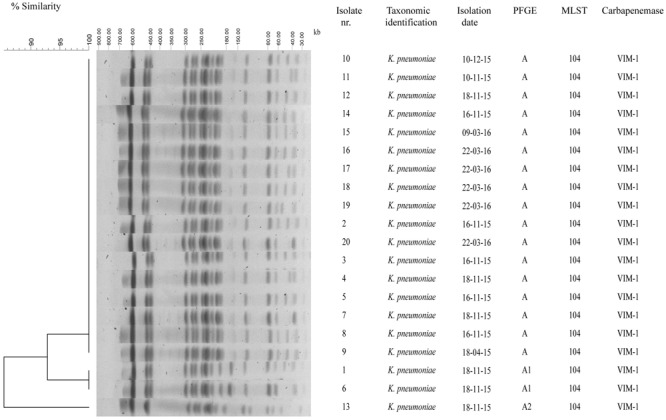
Genotypic analysis of VIM-1 carbapenemase producing *K. pneumoniae* isolates in the NICU. Dendrogram analysis of *Xba*I PFGE profiles of VIM-1 carbapenemase producing *K. pneumoniae* isolates from the NICU. Percentage of similarity and sizes in kilobase (kb) of lambda DNA molecular mass markers are indicated. Isolate number, taxonomic identification, isolation date, PFGE type, MLST, and carbapenemase are shown also.

All CR *K. pneumoniae* isolates from neonates in the NICU showed a MDR phenotype. In fact, they exhibited resistance or intermediate resistance to carbapenem (imipenem, meropenem, ertapenem), resistance to aminopenicillins, ureidopenicillins, third and fourth generation of cephalosporins (ceftazidime, cefotaxime, cefepime), gentamicin, fosfomycin, and trimethoprim–sulfamethoxazole, but were susceptible to amikacin, ciprofloxacin, and colistin (**Table 1**). All *K. pneumoniae* isolates gave a positive result in the MBL-assay performed using the imipenem–EDTA combined disk method. PCR and sequence analysis identified the presence of *bla*_V IM-1_ but not any of the other carbapenemase genes tested in all CR *K. pneumoniae* isolates from NICU (**Figure 2**).

**Table 1:** Antibiotic susceptibility profiles of MDR *K. pneumoniae* in the NICU.

MIC^∗^ (mg/l)			
**Antibiotic**	***K. pneumoniae* (20 total stains)**		
	**MIC_50_**	**MIC_90_**	**Range**
Amoxicillin	>32	>32	>32
Piperacillin–tazobactam	>128	>128	>128
Ceftazidime	>64	>64	>64
Cefotaxime	>64	>64	>64
Cefepime	>64	>64	32 to >64
Imipenem	8	>16	8 to >16
Meropenem	>16	>16	>16
Ertapenem	4	4	4
Fosfomycin	>128	>128	>128
Amikacin	≤2	≤2	≤2
Gentamicin	4	4	4
Ciprofloxacin	0.5	1	0.5–1
Trimethoprim–sulfamethoxazole	>320	>320	>320
Tigecycline	≤0.5	≤0.5	≤0.5
Colistin	≤0.5	≤0.5	≤0.5

*^∗^Minimal inhibitory concentration.*

### Molecular Epidemiology of CR *K. pneumoniae*

**FIGURE 3:**
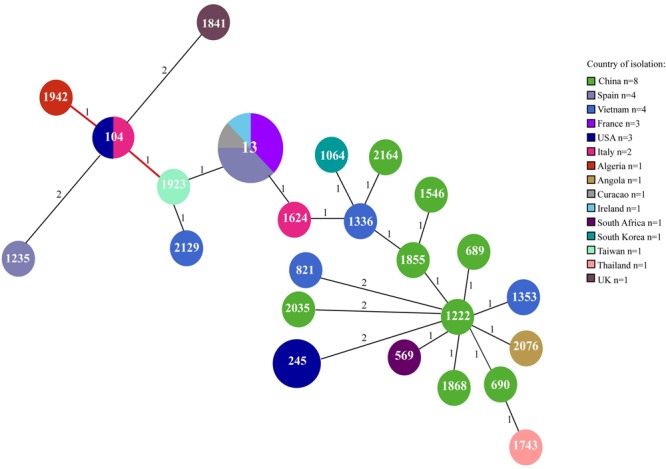
Minimum spanning tree of ST104 and single-locus and double-locus variant isolates at Klebsiella PubMLST (http://bigsdb.pasteur.fr/perl/bigsdb/bigsdb.pl?db=pubmlst_klebsiella_isolates_public&page=profiles). ST1942 and ST1923 are single-locus variant of ST104. The other STs are ST104 double-locus variant. Numbers inside each circle indicate the ST types. The number on the branch indicates the different alleles between STs. Circle size is proportional to the number of isolates belonging to the same ST type. The pie charts for each ST indicate the country of isolation.

To assess whether the increase of CR *K. pneumoniae* isolation in the NICU was caused by the spread of epidemic strains, all 20 CR *K. pneumoniae* isolates from 20 neonates were genotyped by *Xba*I digestion, PFGE, and dendrogram analysis. Molecular analysis identified an identical macrorestriction pattern in 17 isolates, which we named PFGE type A, whereas two isolates showing difference in the migration of one band and one isolate in the migration of four bands and a similarity of >85% at dendrogram analysis were classified into subtypes A1 and A2, respectively (**Figure 2**). The above data indicated that the increase of CR *K. pneumoniae* isolation in the NICU was caused by the spread of a single VIM-1-producing *K. pneumoniae* epidemic genotype. MLST analysis assigned CR *K. pneumoniae* isolates with PFGE type A and subtypes A1 and A2 to ST104 (**Figure 2**). *K. pneumoniae* ST104 isolate from milk during bovine mastitis and *K. pneumoniae* ST1923 and ST1942 isolates from human blood and human feces, respectively, which were single-locus variants of ST104, and 21 other STs, which were double-locus variants of ST104, were reported worldwide and found in Klebsiella PubMLST isolates database^2^ (**Figure 3**).

### Genomic Features of VIM-1-Producing *K. pneumoniae* ST104 KP4898

Additional epidemiological information was provided by genome sequence of representative *K. pneumoniae* KP4898 isolate. Genome sequence confirmed MLST assignment of *K. pneumoniae* KP4898 isolate to ST104. Capsular typing through sequencing of CD1–VR2–CD2 region of wzc and outer membrane protein wzi genes of locus identified wzc-32 and wzi-102 alleles, respectively, which are associated with K31 capsular type. In the KP4898, an array of virulence-associated genes was found, which includes the type-3 fimbriae cluster *mrkABCDF* and transcription regulators *mrkHIJ*, the yersiniabactin siderophore cluster *ybtAPQSTUX*, the yersiniabactin receptor *fyuA*, yersiniabactin biosynthetic protein genes *irp1* and *irp2* and *allB* and *allD* genes of allantoinase cluster. The analysis of drug-associated resistance genes in KP4898 genome identified *bla*_SHV -5_ and *bla*_SHV -12_ extended-spectrum β-lactamases, *bla*_V IM-1_ carbapenemase, *aadA1, aphA15*, and *aacA4* aminoglycoside resistance genes, a macrolide 2′-phosphotransferase cluster, other antimicrobial resistance genes, heavy metal resistance genes, efflux system, and regulators genes (Supplementary Table S2). The PlasmidFinder web tool identified A/C, FII(K) and FIB(K) replicons, thus suggesting the presence of at least three plasmids in KP4898 genome.

### Conjugal Transfer of Carbapenem Resistance

The transfer of carbapenem resistance from VIM-1-producing ST104 *K. pneumoniae* isolates with PFGE type A and subtypes A1 and A2 was evaluated by filter mating experiments. Resistance or intermediate resistance to aminopenicillins, ureidopenicillins, third and fourth generation cephems, imipenem, but not meropenem and ertapenem, was transferred from ST104 *K. pneumoniae* isolates with PFGE type A and subtypes A1 and A2 to *E. coli* J53 aziR at frequency ranging from 6.5 × 10^-3^ to 1.5 × 10^-3^ cfu/recipient cells. The frequency of transfer of imipenem resistance from *K. pneumoniae* ST104/PFGE A, A1, and A2 isolates to *E. coli* J53 aziR did not change if transconjugants were selected in the presence of imipenem and sodium azide or ampicillin and sodium azide. All transconjugants showed identical antimicrobial susceptibility profile and resistance to sodium azide (**Table 2**). Moreover, all transconjugants showed a PFGE profile identical with that of the recipient strain. The presence of *bla*_V IM-1_ and *bla*_SHV -12_ genes was demonstrated in all transconjugants. Furthermore, PBRT identified A/C replicon and IncA/C incompatibility group plasmid/s in ESBL positive and CR *K. pneumoniae* ST104 donor isolates and *E. coli* transconjugants expressing *bla*_V IM-1_ and *bla*_SHV -12_ genes. The above data suggested that conjugative plasmid/s mediated the horizontal transfer of carbapenem resistance.

**Table 2:** Conjugative transfer of carbapenem resistance.

MIC (mg/l)			
**Antibiotic**	**Strain**		
	***K. pneumoniae* ST104/A-A2**	***E. coli* J53 aziR (p1KPST104)**	***E. coli* J53 aziR**
Amoxicillin	>32	>32	4
Piperacillin–tazobactam	>128	>128	2
Ceftazidime	>64	8	0.25
Cefotaxime	>64	>64	≤0.25
Cefepime	>64	2	≤0.25
Imipenem	8	8	0.25
Meropenem	>16	≤0.25	≤0.25
Ertapenem	4	≤0.5	≤0.5
Fosfomycin	>128	≤16	≤16
Amikacin	≤2	≤2	≤2
Gentamicin	4	≤1	≤1
Ciprofloxacin	0.5	≤0.25	≤0.25
Trimethoprim–sulfamethoxazole	>320	≤20	≤20
Tigecycline	≤0.5	≤0.5	≤0.5
Colistin	≤0.5	≤0.5	≤0.5

### Genetic Structure of pIncAC-KP4898

**FIGURE 4:**
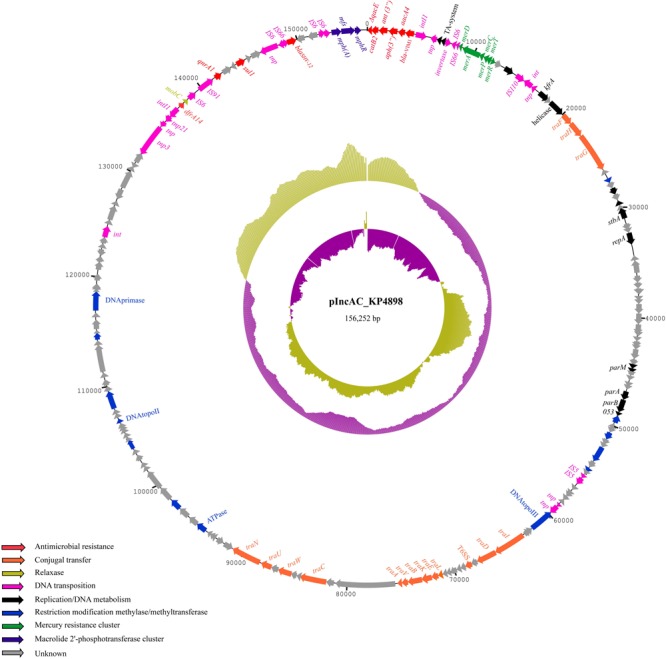
Schematic map of pIncAC_KP4898 plasmid. Circular map of pIncAC_KP4898. The outermost ring represents the pIncAC_KP4898 plasmid with arrows indicating genes/ORFs involved in the various cellular pathways. Functions of the genes and ORFs found are color coded and shown in the left bottom corner of the figure. The middle ring and the innermost ring represent a GC plot and a GC skew plot, respectively. For both plots, magenta and olive green indicate the measures below and above the average, respectively.

**FIGURE 5:**
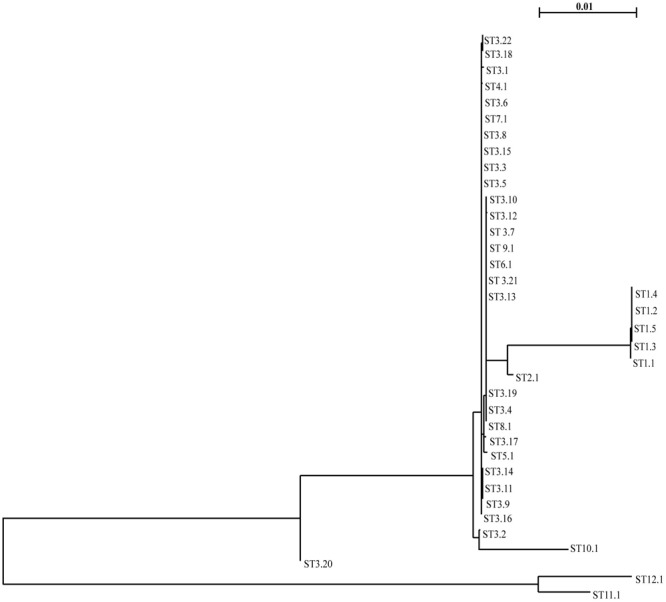
Phylogeny of cgPMLST IncA/C plasmids. Neighbor-joining phylogenetic trees based on multiple alignments of concatenated alleles of the 28 IncA/C conserved genes. The bar at the top of the figure shows the amount of genetic change corresponding to the length of each branch.

In the genome sequence of *E. coli* transconjugants, one single plasmid was identified, which was present in *K. pneumoniae* KP4898 donor strain, but not in *E. coli* J53 recipient strain, and was designated pIncAC-KP4898, for plasmid of IncA/C incompatibility group from *K. pneumoniae* 4898 isolate. The pIncAC-KP4898 plasmid was 156,252 bp in size, with an average G + C content of 52.6%. Genome annotation identified 190 open reading frames (ORFs), of which 145 were transcribed in a clockwise orientation, while the remaining 45 were transcribed counterclockwise. Of these ORFs, 14 were associated with plasmid DNA replication and partition, 16 with DNA transfer, 13 with DNA-restriction and site-specific DNA methylation, 25 with DNA transposition, 19 with antimicrobial resistance, and 103 with unknown functions (**Figure 4** and Supplementary Table S3). The pIncAC-KP4898 scaffold included the *repA, parA, parB*, and *053* genes, *parM, kfrA*, and a putative toxin–antitoxin system, which were demonstrated to be important for maintenance and replication of IncA/C plasmid (Hancock et al., 2017). Based on RepA similarity (Carattoli et al., 2006), the pIncAC-KP4898 plasmid belongs to IncA/C1 group. The IncA/C PMLST scheme based on the *repA, parA, parB*, and *053* genes and the cgPMLST scheme that extend the 4-gene PMLST to 28 conserved genes (Hancock et al., 2017) assigned pIncAC-KP4898 to novel profiles, which corresponded to ST12 and ST12.1, respectively. The phylogeny of IncA/C 28-gene profiles showed that ST12.1 was more closely related to ST11.1, to which IncA/C1 group pRA1 plasmid (GenBank accession number FJ705807.1) has been assigned, than to the remaining 10 profiles (ST1 to ST10), which corresponded to IncA/C2 group plasmids (**Figure 5**).

Plasmid pIncAC-KP4898 contained a composite transposon of 25,716 bp (residues 139,120–156,252 and 1–8,583, G + C content: 57.7%) with four IS6 family transposase and 14 bp inverted repeats (TTTGCAACAGTGCC) at residues 152,072–152,086 and 8,569–8,583 (3′-flanking region) (**Figure 4**). Within this transposon lied a class 1 integron containing a 5′-conserved structure (CS) with *int1* site-specific integrase, and five head-to-tail arranged gene cassettes consisting of the genes *bla*_V IM-1_, *aacA4* family aminoglycoside N(6′)-acetyltransferase gene, aminoglycoside *O*-phosphotransferase *APH(3′)-XV* encoding gene, ANT(3″) family aminoglycoside nucleotidyl transferase gene, type B-2 chloramphenicol *O*-acetyltransferase *catB2* gene. The 3′-CS showed the *qacED1*-encoding gene, which confers resistance to quaternary ammonium compounds. The Tn3-like composite transposon included also the *bla*_SHV -12_ gene flanked by IS6 family transposases in inverted orientation, a macrolide 2′-phosphotransferase gene cluster consisting of macrolide 2′-phosphotransferase *mph(A)*, transporter *mfs* and macrolide 2′-phosphotransferase I repressor A *mphR* genes, quinolone resistance pentapeptide repeat protein *qnrA1* gene, and sulfonamide-resistant dihydropteroate synthase *sul1* genes. A mercury resistance gene clusters and trimethoprim-resistant dihydrofolate reductase *dfrA14* gene were carried by pIncAC-KP4898 apart from the identified transposon region. In addition, 16 genes encoding transfer functions and a conjugative apparatus were found in pIncAC-KP4898, which indicated that the plasmid was self-conjugative (**Figure 4** and Supplementary Table S3).

## Discussion

The emergence of multidrug-resistant and carbapenem-resistant microorganisms has become an alarming phenomenon in children and neonates (Logan, 2012; Logan et al., 2015). Intestinal carriage of carbapenemase producing Enterobacteriaceae is an important reservoir and source of dissemination of resistance to carbapenems among Gram-negative bacteria in the community and in the hospital setting (Nordmann and Poirel, 2014; Viau et al., 2016; Logan and Weinstein, 2017).

The current study investigates the molecular epidemiology and the genetic mechanism of acquisition of carbapenem resistance of multidrug-resistant *K. pneumoniae* isolated into two consecutive clusters from rectal swabs of 20 neonates in the NICU of the V. Monaldi Hospital in Naples, Italy. Our data showed the selection of a single VIM-1-producing *K. pneumoniae* epidemic genotype assigned to PFGE type A, A1, and A2 and ST104, which was isolated only from patients in the NICU but not in other wards of the hospital, thus suggesting that cross-transmission among intestinal carrier neonates may have been favored the spread of VIM-1 producing *K. pneumoniae* epidemic clone. The diffusion of VIM-1 producers *K. pneumoniae* is uncommon in Italy, where the vast majority of carbapenemase producers *K. pneumoniae* are KPC producers (Giani et al., 2017; Grundmann et al., 2017). After the first isolation of ST104 *K. pneumoniae* from milk during bovine mastitis (Paulin-Curlee et al., 2007), sporadic isolations of ST104 (Baraniak et al., 2013; Maatallah et al., 2014; Esteban-Cantos et al., 2017), were reported from human infections/colonizations. Also, *K. pneumoniae* ST1923 and ST1942, which were single-loci variants of ST104, were isolated from human blood and human feces (Yan et al., 2015), and *K. pneumoniae* isolates assigned to 21 other STs, which were double-locus variants of ST104, were isolated from difference sources including clinical specimens (**Figure 3**). Since ST104 *K. pneumoniae* human isolates were either ESBL producing (Baraniak et al., 2013; Maatallah et al., 2014; Esteban-Cantos et al., 2017) or carbapenemase producing (Esteban-Cantos et al., 2017), we can hypothesize that antimicrobial resistance might have selected ST104 *K. pneumoniae* isolates among bacterial population.

In accordance with this, data reported herein showed that *K. pneumoniae* isolates from neonates in the NICU showed a MDR phenotype, being resistant to all classes of β-lactams including third and fourth generation cephems and carbapenems, fosfomycin, gentamicin, and trimethoprim–sulfamethoxazole, but susceptible to quinolones, amikacin and colistin. This is particularly alarming in neonates, for which limited options of antimicrobial therapy are available. Also, genome sequence of VIM-1-producing *K. pneumoniae* ST104 KP4898 showed the presence of virulence-associated genes and antimicrobial resistance genes (Supplementary Table S2). Interestingly, ST104 *K. pneumoniae* isolates from neonates in the NICU showed resistance to antimicrobials frequently used in neonates, such as third and fourth generation cephems, carbapenems, gentamicin, and trimethoprim–sulfamethoxazole, while they retain susceptibility to fluoroquinolones, which are not recommended in this clinical setting. Since it has been demonstrated that previous combination antimicrobial treatment with ampicillin and gentamicin is independent risk factors for acquisition of extended-spectrum β-lactamase-producing *K. pneumoniae* and *Serratia marcescens* in neonates (Crivaro et al., 2007), we can speculate that resistance to third and fourth generation cephems and carbapenems might have been selected by their frequent use in the NICU.

Several studies demonstrate that the horizontal gene transfer through conjugative plasmids and transposons contributes to the spread of resistance to carbapenems (Bialek-Davenet et al., 2014; Nordmann and Poirel, 2014; Viau et al., 2016). Accordingly, we showed that resistance to aminopenicillins, ureidopenicillins, third and fourth generation cephems, imipenem, but not meropenem and ertapenem, was transferred from VIM-1-producing ST104 *K. pneumoniae* isolates assigned to either PFGE type A, A1, or A2 to susceptible *E. coli* along with a *bla*VIM-1 positive plasmid of IncA/C1 incompatibility group and 156,252 bp in size, which we named pIncAC_KP4898 (**Figure 4**). Based on the above data, we postulate that imipenem resistance depends mainly on the expression of VIM-1 carbapenemase carried by pIncAC_KP4898, while meropenem and ertapenem resistance might be contributed by additional resistance mechanisms in *K. pneumoniae* isolates, such as altered permeability due to changes in the expression of porins or efflux systems (Goodman et al., 2016). Further experiments will be necessary to validate such hypothesis. The pIncAC-KP4898 scaffold was assigned to novel ST12 and ST12.1 profiles. Interestingly, it resulted to be more closely related to IncA/C1 group pRA1 plasmid from the fish pathogen *Aeromonas hydrophila* (Fricke et al., 2009) assigned to ST11.1, than to the majority of IncA/C plasmid belonging to IncA/C2 group and ST1 to ST10 profiles (**Figure 5** and **Supplementary Figure S1**), which are associated with the carriage of *bla*_NDM_ or *bla*_CMY_ (Hancock et al., 2017). While InCA/C2 group plasmids were frequently found in Enterobacteriaceae isolated from human and non-human sources (Hancock et al., 2017), only two IncA/C1 group complete plasmid sequences are available in GenBank, pRA1 plasmid from *A. hydrophila* environmental isolate (Fricke et al., 2009) and pIncAC_KP4898 plasmid from ST104 *K. pneumoniae* clinical isolate described in this study. Of these, pIncAC_KP4898 is the first InCA/C1 plasmid carrying *bla*_V IM-like_ sequences. Similarly to IncA/C1 group pRA1 plasmid (Fricke et al., 2009) and several IncA/C 2 group plasmids (Hancock et al., 2017), pIncAC-KP4898 carried several *tra* genes encoding transfer functions and was self-conjugative.

pIncAC_KP4898 plasmid presents a composite transposon of approximately 26 kb, which includes *bla*_SHV -12_ extended spectrum β-lactamase gene, flanked by IS6 elements and a class I integron with *bla*_V IM-1_ carbapenemase gene, *aacA4* family aminoglycoside N(6′)-acetyltransferase gene, aminoglycoside *O*-phosphotransferase *APH(3′)-XV* encoding gene, ANT(3″) family aminoglycoside nucleotidyl transferase gene, type B-2 chloramphenicol *O*-acetyltransferase *catB2* gene. A class I integron showing identical gene cassettes array is found in IncN plasmid pOW16C2 from *K. pneumoniae* environmental isolate (Zurfluh et al., 2015), and non-typeable plasmids pAX22 from *Achromobacter xylosoxidans* (Di Pilato et al., 2014) and plasmid of >300 kb from *E. coli* strain W1058 (GenBank accession number KF856617; Porres-Osante et al., 2014). Based on the above all data, we can postulate that genetic structure of class I integron with *bla*_V IM-1_ carried by pIncAC-KP4898 might have an environmental source. In accordance with our data, VIM-1 has been shown to be the most prevalent allele variants among VIM-producing isolates, having global geographical distribution and being isolated in multiple Enterobacteriaceae species (Matsumura et al., 2017). Moreover, *Aeromonas caviae* carrying *bla*_V IM-1_ and *bla*_V IM-35_ inside class I integrons were isolated from clinical surveillance cultures in Israeli hospitals (Adler et al., 2014); *A. caviae* carrying *bla*_V IM-1_ and *bla*_SHV -12_ into a transferable plasmid were isolated from the blood cultures of 1-day-old newborn in Florence, Italy (Antonelli et al., 2016). A macrolide 2′-phosphotransferase gene cluster inside the composite transposon and a mercury resistance gene clusters and trimethoprim-resistant dihydrofolate reductase *dfrA14* gene outside the transposon region were additional resistance genes, which might have contributed to the selection of pIncAC_KP4898 into ST104 *K. pneumoniae*.

## Conclusion

The spread of carbapenem resistance in *K. pneumoniae* from neonates in the NICU was due to the acquisition of plasmid pIncAC-KP4898, carrying the *bla*_V IM-1_ gene and several additional resistance genes into the scaffold of an IncA/C1 group self-conjugative plasmid. The composite genetic structure of pIncAC-KP4898 might have been generated by the acquisition of different regions from different sources mediated by multiple recombination events.

## Author Contributions

DS and RZ conceived the study and participated in its design and coordination. VC collected the epidemiological data and performed the infection control in the hospital. MB and SC collected the microbiological data. EE, MDF, FP, and MT performed laboratory analyses. SG, PM, DS, and RZ performed data analyses. EE, SG, DS, and RZ wrote the manuscript. All authors read and approved the final manuscript.

## Conflict of Interest Statement

The authors declare that the research was conducted in the absence of any commercial or financial relationships that could be construed as a potential conflict of interest.

## Footnotes

1 http://bigsdb.pasteur.fr/perl/bigsdb/bigsdb.pl?db=pubmlst_klebsiella_seqdef_public&page=sequenceQuery

2 http://bigsdb.pasteur.fr/perl/bigsdb/bigsdb.pl?db=pubmlst_klebsiella_isolates_public&page=profiles

## Supplementary Material

FIGURE S1 Minimum spanning tree showing the IncA/C core gene PMLST (cgPMLST). Numbers inside each circle are the cgST types. The size of the circle indicates the number of the isolates belonging to the same cgST type. The number on the branch indicates the different alleles between cgSTs.
