# Supplementary figures and images for: A Novel IncA/C1 Group Conjugative Plasmid, Encoding VIM-1 Metallo-Beta-Lactamase, Mediates the Acquisition of Carbapenem Resistance in ST104 Klebsiella pneumoniae Isolates from Neonates in the Intensive Care Unit of V. Monaldi Hospital in Naples
Source: Front Microbiol. 2017 Nov 3;8:2135. doi: 10.3389/fmicb.2017.02135 (PMC5675864; doi:10.3389/fmicb.2017.02135)

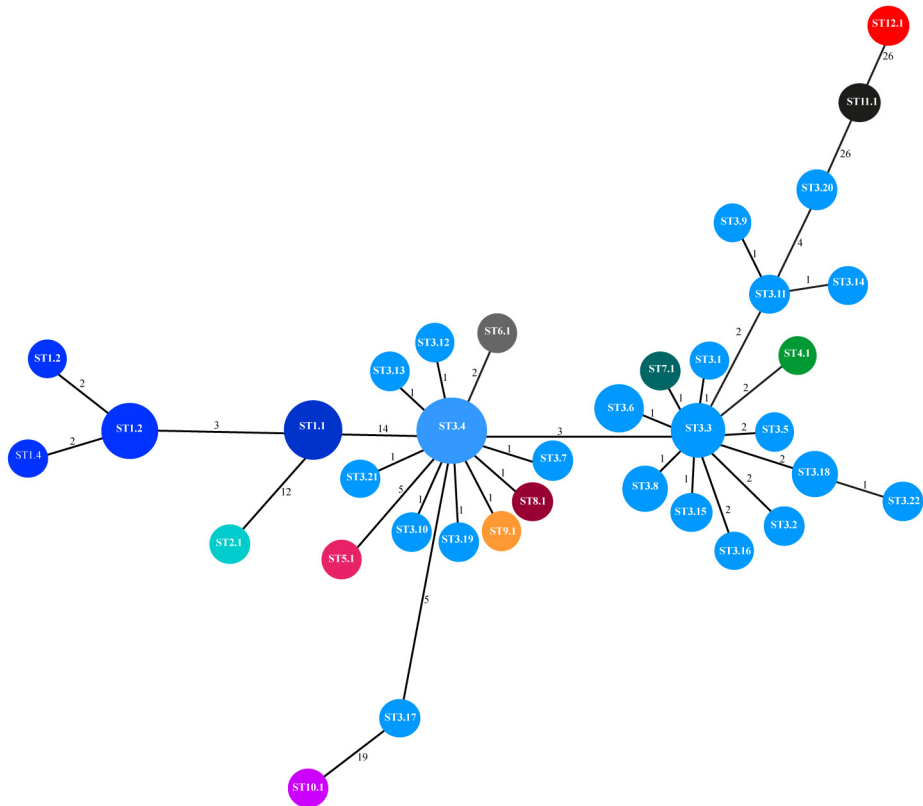

Supplement: FIGURE S1 — Minimum spanning tree showing the IncA/C core gene PMLST (cgPMLST). Numbers inside each circle are the cgST types. The size of the circle indicates the number of the isolates belonging to the same cgST type. The number on the branch indicates the different alleles between cgSTs. [file Image_1.pdf]
