# Supplementary material for: A Novel IncA/C1 Group Conjugative Plasmid, Encoding VIM-1 Metallo-Beta-Lactamase, Mediates the Acquisition of Carbapenem Resistance in ST104 Klebsiella pneumoniae Isolates from Neonates in the Intensive Care Unit of V. Monaldi Hospital in Naples
Source: Front Microbiol. 2017 Nov 3;8:2135. doi: 10.3389/fmicb.2017.02135 (PMC5675864; doi:10.3389/fmicb.2017.02135)
Supplement: Supplementary file 2 [file Table_1.DOCX]

**Table S1.** Oligonucleotides used in the study.

| Primer for plasmid’s PCR mapping^a^ | Sequence (5’to 3’) |
| --- | --- |
| 1Rev620 | GTCATCGAGGAAGGTAGCGGAC |
| 2Fw10658 | GTATGTCTGGACCGCAACCA |
| 2Rev349 | CACGCCGGATCAGAACGTAT |
| 4Fw3577 | AATGACGACCTCTGCTTCCG |
| 4Rev263 | TGCTCACAGCCAAACTATCAGG |
| 4Rev458 | GGCCGCTGGAACAAATCAAGGA |
| 4Rev3577 | CGGAAGCAGAGGTCGTCATT |
| 5Fw3390 | GGTTCAGGCTTGGTCCTTACA |
| 5Rev226 | CAGGGTGGCTAACAGGGAGA |
| 6Rev426 | ACCATCCCGATACTTGCGTC |
| 6Rev356 | CGAGACGATACTCCGCCAAT |
| 6Rev588 | CAACTCCTCCAGGCACATGCGT |
| 7Fw1801 | CGTTTTCGAGGACACGGCT |
| 9Fw1085 | GAAGGCACGAACCCAGTTGA |
| 9Rev186 | TAGGGCAGCGCAAGTCAATC |
| 22Fw653 | GGTATTGAGGTGATGCGTGC |
| 22Rev367 | TGTTACGACGGGAGGAGAGA |

^a^ All PCRs included an initial denaturation step of 3 min at 94°C, 35 cycles of denaturation, annealing and extension amplification consisting of 1 min at 94°C, 1 min at 60°C and 2 min at 72°c with 5 min at 72°C for the final extension.

| Primer used to amplify VIM allele | Sequence (5’to 3’) |
| --- | --- |
| 5’VIM1 | ATGTTAAAAGTTATTAGTAGTT |
| 3’VIM1 | CTACTCGGCGACTGAGCGATT |

^a^ The PCR included an initial denaturation step of 3 min at 94°C, 35 cycles of denaturation, annealing and extension amplification consisting of 1 min at 94°C, 1 min at 57°C and 1 min at 72°c with 5 min at 72°C for the final extension.
